# Fast set-based association analysis using summary data from GWAS identifies novel gene loci for human complex traits

**DOI:** 10.1038/srep32894

**Published:** 2016-09-08

**Authors:** Andrew Bakshi, Zhihong Zhu, Anna A. E. Vinkhuyzen, W. David Hill, Allan F. McRae, Peter M. Visscher, Jian Yang

**Affiliations:** 1 Queensland Brain Institute, The University of Queensland, Brisbane, Queensland 4072, Australia; 2 Centre for Systems Genomics, School of BioSciences, The University of Melbourne, Parkville 3010, Victoria, Australia; 3 Institute for Molecular Bioscience, The University of Queensland, Brisbane, Queensland 4072, Australia; 4 Centre for Cognitive Ageing and Cognitive Epidemiology, University of Edinburgh, 7 George Square, Edinburgh, UK; 5 Department of Psychology, University of Edinburgh, Edinburgh, UK; 6 The University of Queensland Diamantina Institute, The Translation Research Institute, Brisbane, Queensland, Australia

## Abstract

We propose a method (fastBAT) that performs a fast set-based association analysis for human complex traits using summary-level data from genome-wide association studies (GWAS) and linkage disequilibrium (LD) data from a reference sample with individual-level genotypes. We demonstrate using simulations and analyses of real datasets that fastBAT is more accurate and orders of magnitude faster than the prevailing methods. Using fastBAT, we analyze summary data from the latest meta-analyses of GWAS on 150,064–339,224 individuals for height, body mass index (BMI), and schizophrenia. We identify 6 novel gene loci for height, 2 for BMI, and 3 for schizophrenia at *P*_fastBAT_ < 5 × 10^−8^. The gain of power is due to multiple small independent association signals at these loci (e.g. the *THRB* and *FOXP1* loci for schizophrenia). The method is general and can be applied to GWAS data for all complex traits and diseases in humans and to such data in other species.

Due to the polygenic nature of most human complex traits and diseases, the effect sizes of individual genetic variants are usually very small, limiting the statistical power to detect them, even in large samples^1^. Emerging evidence have suggested that disease- or trait-associated genetic variants identified from genome-wide association studies (GWAS) tend to be in enriched genic regions^2^,^3^, and often there are multiple associated variants at a single locus^4^,^5^. Therefore, for the discovery of complex trait genes, it would be more powerful to test the aggregated effect of a set of SNPs (e.g. SNPs within or close to a gene) using a set-based association approach, e.g. the set-test method implemented in PLINK (–set option)^6^. In a meta-analysis of GWAS, however, individual-level genotype data are usually not available. Liu *et al*.^7^ developed a simulation-based approach, called VEGAS (Versatile Gene-based Association Study), which implements a gene-based association test using summary data from a GWAS or meta-analysis and linkage disequilibrium (LD) between SNPs from HapMap^8^ or 1000 Genome Project (1 KGP)^9^ reference panels. VEGAS is much faster than the permutation-based approach in PLINK^6^, and does not require individual-level genotype data. However, both VEGAS and PLINK use resampling-based approaches, which have two main limitations. Firstly, the lower bound of a p-value is constrained by the number of permutations or simulations (*s*), such that the minimum p-value is 1/*s*. Secondly, the resampling-based methods are computationally demanding as a consequence of a large number of permutations or simulations. While the simulation-based approach (i.e. VEGAS) is significantly less resource-intensive, both VEGAS and PLINK have computing efficiency inversely proportional to *s*. There are several powerful and/or efficient methods that have been recently developed^10^,^11^,^12^,^13^. For example, GATES is a “best-SNP picking” method, which is more powerful than the “all-SNP aggregating” methods (e.g. VEGAS) for a relatively simple genetic architecture (GATES is less powerful than VEGAS if the trait is highly polygenic)^10^. aSPUs is an adaptive approach that combines multiple SPU (Sum of powered score) tests for summary data with aSPUs(infinite) being similar to GATES and aSPUs(2) being similar to VEGAS but it requires simulations to calculate p-values^11^. HYST is hybrid approach that aggregates the selected best SNPs in different LD blocks and thus gains power over GATES^13^. HYST is particularly powerful in detecting genes involved in the same protein-protein interaction pathways. GATES and HYST are implemented in a software tool called KGG with an elegant graphical user interface (although it is challenging to run a large number of analyses). Both methods are computationally fast (they only require summary-level data and do not need permutation or simulation for significance test). There is another tool called Pascal which shows a significant improvement in speed over Pascal^12^. In this study, we proposed fastBAT, a fast and flexible set-Based Association Test, which overcomes the limitations of the resampling-based methods by calculating the p-value for a set of SNPs directly from an approximated distribution with a significant improvement of computational efficiency over existing methods. We further developed a LD-pruned fastBAT approach which gains power if the causal variants for a complex trait are enriched in genomic regions with lower LD than average. We demonstrated the efficiency, accuracy and power of fastBAT using simulations, and identified novel associations using real data from the latest meta-analyses of GWAS for height, body mass index (BMI), and SCZ.

## Results

### Comparing fastBAT with the prevailing methods

**Figure 1:**
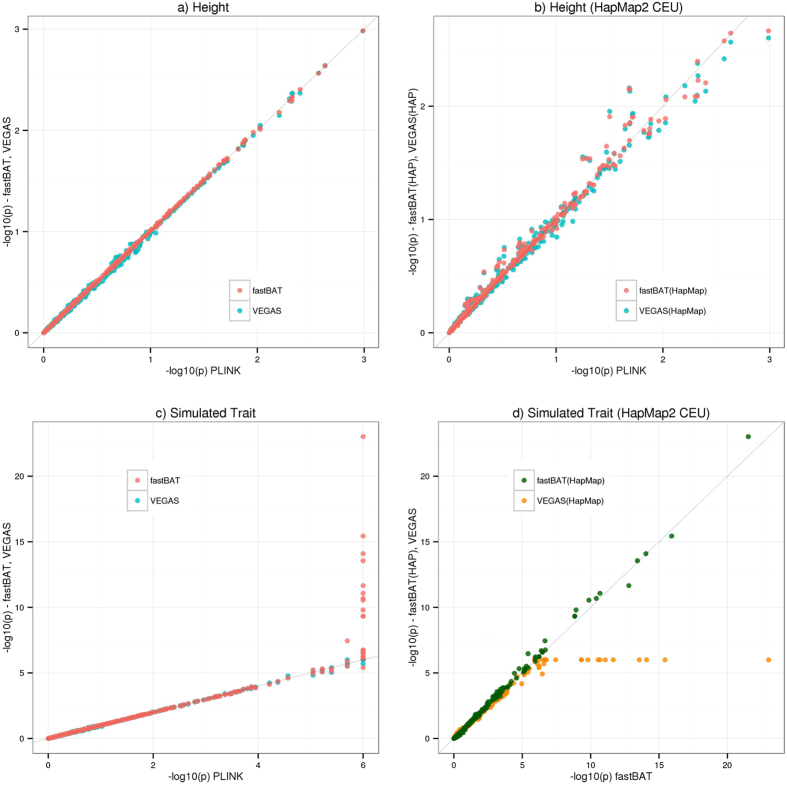
Comparison of fastBAT, VEGAS and PLINK. Shown on all the panels are the −log10(p-value) from the set-based analysis of 583 genes using genotypes of 7,608 SNPs on chromosome 22 in the ARIC study (*n* = 7,661) and height or simulated phenotype data (Methods). A gene is defined as all SNPs within 50 Kb of UTRs of a gene. In panels (a–c), the x-axis represents −log10(p-value) from the PLINK analysis using individual-level genotype and phenotype data. The fastBAT and VEGAS analyses use summary data from GWAS for height or the simulated phenotype. In panels (a,c), shown on the y-axis are the results from the fastBAT and VEGAS analyses using LD from the ARIC data. In panels (b,d), the fastBAT and VEGAS analyses are based on LD from HapMap2 CEU panel (*n* = 90). In panel (d), shown on the x-axis is the −log10(p-value) from fastBAT with LD from the ARIC data.

Details of the fastBAT method can be found in Methods. In brief, fastBAT calculates the association p-value for a set of SNPs (e.g. SNPs ±50 Kb of a gene) from an approximated distribution of the sum of *χ*^2^-statistics over the SNPs using summary data from GWAS and LD correlations between SNPs from a reference sample with individual-level genotypes. We compared fastBAT with VEGAS and PLINK using real genotype data (*n* = 7,661 unrelated individuals and *m* = 7,608 SNPs on chromosome 22) with real and simulated phenotypes in two scenarios (Methods): I) when individual-level genotype data are available (summary statistics and LD calculated from the same sample); II) when individual-level genotype data are unavailable (LD data from HapMap phase 2, HapMap2, CEU panel^8^). We first investigated the behavior of the fastBAT p-value under the null hypothesis of no SNP-trait associations and did not observe any inflation or deflation (Supplementary Fig. 1). Results from the analysis of real height phenotype show that when the individual-level genotype data were available (Fig. 1a), fastBAT was almost identical to PLINK, with a squared correlation of −log10 p-value of *r*^*2*^ = 0.9998 between the two (the regression intercept is nearly zero). The correlation between PLINK and VEGAS was slightly smaller (*r*^2^ = 0.9989) due to the limited number of simulations in the initial run for VEGAS (by default VEGAS runs 1000 simulations initially, which is then followed by 1 million simulations for genes with p-value < 0.001 in the initial run) but the difference was negligible. For the analysis with real height phenotype data in scenario II (Fig. 1b), there was an expected loss of precision in p-values between PLINK and fastBAT (*r*^*2*^ = 0.9862) because of the use of LD from HapMap2 CEU panel to approximate that in the ARIC data. Results from the analysis of the simulated trait in scenario I (Fig. 1c) also show strong concordance between PLINK and fastBAT/VEGAS for p-values >10^−6^, consistent with the result from the analysis using Pascal-Sum^12^. While the smallest possible p-values in PLINK/VEGAS were constrained by the 10^6^ permutations/simulations upper bound, fastBAT was free of such limitation. We have shown in Fig. 1a that if individual-level genotypes are available, fastBAT is equivalent to PLINK. Since it is computationally infeasible to calculate p-values using PLINK with >10^6^ permutations, we used fastBAT as a benchmark for comparison for the simulated trait in scenario II. We observed a strong concordance between the fastBAT results with LD from the ARIC and HapMap2 CEU data (*r*^*2*^ = 0.9866), despite the sample size of the ARIC cohort (*n* = 7,661) being >80 times larger than that of HapMap2 CEU (*n* = 90). This suggests that the set-based test is robust to sampling errors in LD estimation^10^,^13^.

Furthermore, we show using simulations of unlinked SNPs (Methods) that if there is only one causal variant, the p-value from a set-based test is expected to be larger (less significant) than that of the top associated SNP from single-SNP based tests (Supplementary Fig. 2). The gain of power for a set-based test is mainly due to the smaller number of tests as compared with single-SNP based GWAS, e.g. for set-based tests at gene regions (also known as a gene-based test), the maximum number of tests is <20,000 regardless of the total number of SNPs in the data. The set-based test gains power if there are multiple causal variants in the set (or more precisely, SNPs in the set are in LD with multiple causal variants) as demonstrated by our simulation results (Methods and Supplementary Fig. 2). We will show below the gain of power in real data analysis due to multiple signals at single loci.

### The gain of power by removing SNPs in high LD

**Figure 2:**
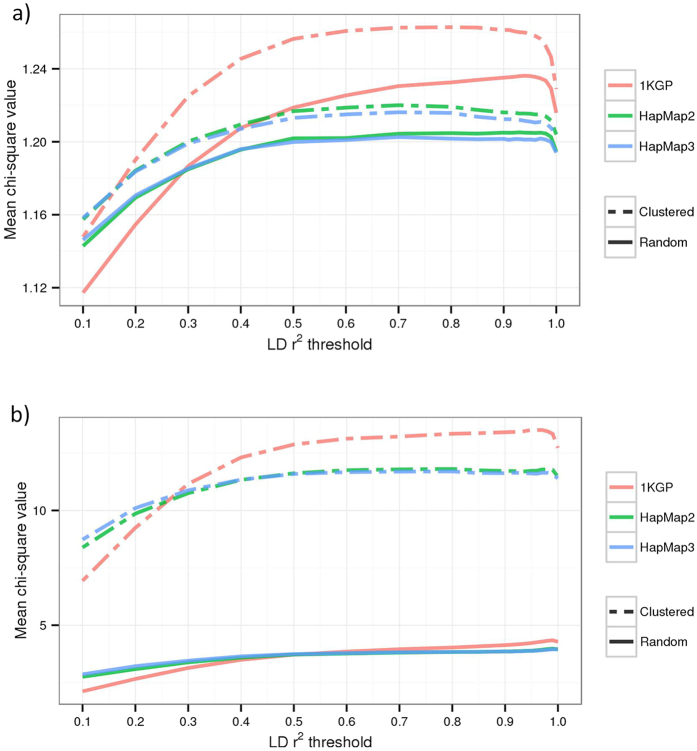
Power of the LD-pruned fastBAT as a function of *r*^2^ threshold used for LD pruning. Shown are the results from simulations based on WGS data from the UK10K project (n = 3,781) under two scenarios, i.e. causal variants clustered or randomly distributed (Methods). The LD-pruned fastBAT analysis is performed at a range of thresholds for LD pruning (shown on x-axis) using common SNPs (MAF ≥ 0.01) on three different panels, i.e. all sequence variants, SNPs on HapMap2 and SNPs on HapMap3. The power is measured by mean *χ*^2^_1_value of all genes (panel a) or genes harboring at least one of the simulated causal variants (panel b), where *χ*^2^_1_ is calculated from *P*_fastBAT_. Each plotted value is an average from 500 simulations.

Previous study suggests that the set-based association analysis approaches such as that implemented in PLINK lose power if there are SNPs in extremely high LD in the set^14^. We found in simulations (Methods) that a set-based approach gained power if there were SNPs in perfect LD with the causal variants, and lost power if there were SNPs in perfect LD with null markers (Supplementary Fig. 3), where null markers are defined as SNPs that are independent from the causal variants. These results suggest that power can be gained by pruning SNPs that are in extremely high LD (e.g. LD *r*^2^ > 0.9) in particular if the causal variants tend to be enriched in genomic regions with lower LD^15^. We therefore developed a LD-pruned fastBAT method (Methods). We demonstrate using simulations (Methods) that the LD-pruned (e.g. using a LD *r*^2^ threshold of 0.9 or 0.99) fastBAT method is slightly more powerful than the original fastBAT method in two different simulation scenarios (causal variants were either randomly distributed or clustered in small regions) (Supplementary Table 1). We re-ran the fastBAT-pruning analysis with a range of threshold *r*^2^ values and found that the LD-pruned fastBAT achieved the largest power gain at *r*^2^ threshold from approximately 0.9 to 0.99 depending on the SNP panel (HapMap2, HapMap3 or whole genome sequencing) and genetic architecture of the trait (Fig. 2). In practice, we recommend a threshold *r*^2^ value of 0.9 regardless of SNP panel, and do not recommend a threshold *r*^2^ < 0.7. In addition, we did not observe any inflation in −log10(p-value) for the LD-pruned fastBAT method under the null hypothesis that there was no genetic effect (Supplementary Fig. 4).

### Novel gene loci for height, body mass index (BMI) and schizophrenia (SCZ)

We applied fastBAT to summary data from the latest meta-analyses of GWAS for height^5^, BMI^16^ and SCZ 17 (Methods). We performed a gene-based test, where a SNP set was defined as the SNPs within ±50 Kb away from the UTRs of a gene. We used the genotype data from the Health Retirement Study (HRS) as the reference for LD estimation and used a LD *r*^2^ threshold value of 0.9 for LD pruning within a set. We identified 50 novel genes loci for height, 8 for BMI and 29 for abbreviated SCZ at a genome-wide significance level (i.e. *P* < 2 × 10^−6^ where the threshold was calculated as 0.05 divided by the number of tests for each trait) (Supplementary Table 4). A novel gene discovery was defined as a gene that passed genome-wide significance level (*P*_fastBAT_ < 2 × 10^−6^) in the gene-based analysis and there was no genome-wide significant SNP (*P*_GWAS_ > 5 × 10^−8^) within ±1 Mb of the gene. We hypothesize that the reason why the SNPs at these gene loci did not reach genome-wide significance level in GWAS is because of the lack of power, although the sample sizes of those studies were very large (Supplementary Table 2), and we predict that these genes will be discovered by GWAS with larger sample sizes in the future. This hypothesis is supported by the evidence from our analyses using the earlier versions of the GWAS summary data (Supplementary Table 3 and Methods), where we performed a gene-based fastBAT analysis using the earlier version of GWAS summary data for height and SCZ (Supplementary Table 3) and identified 19 “novel genes” for height and 4 for SCZ, all of which reached genome-wide significance level (*P*_GWAS_ < 5 × 10^−8^) in the latest GWAS. We then performed the same analysis using fastBAT without LD pruning, Pascal-Sum and Pascal-Max, and counted the number of replicated “novel” genes as above (Supplementary Table 5). We found that fastBAT with LD pruning at an *r*^2^ threshold of 0.9 (the default method in the GCTA-fastBAT software tool) discovered the largest number of replicated “novel” genes.

### The gain of power due to multiple associated signals at single loci

**Figure 3:**
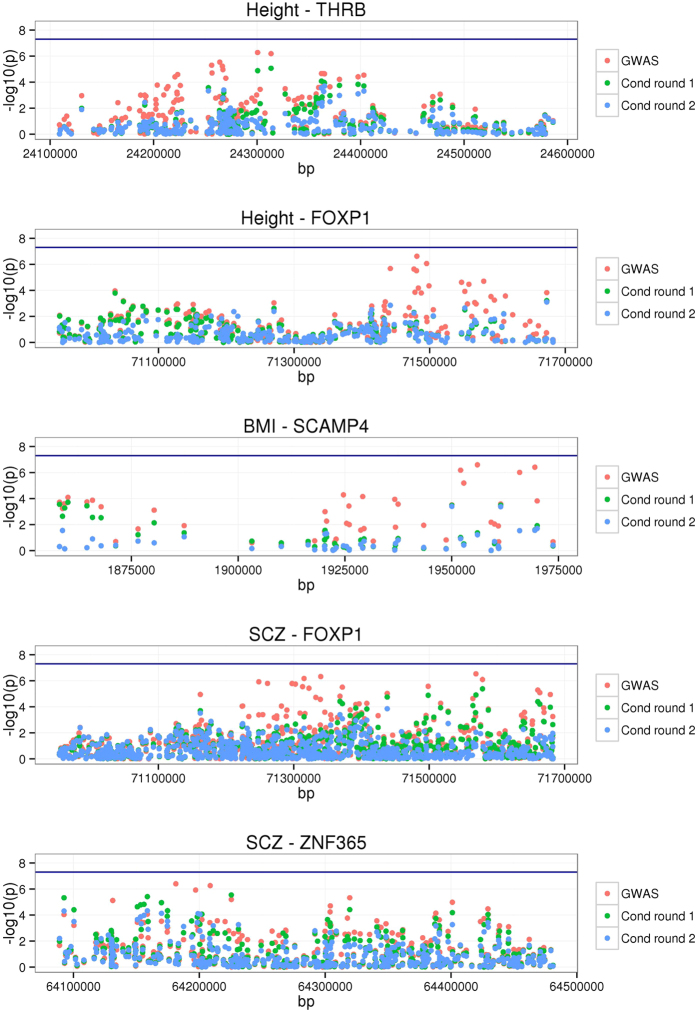
Conditional association analysis at the novel gene loci for height, BMI and schizophrenia. In the analysis of the latest GWAS data for height, BMI and schizophrenia, there are 5 genes loci at which the signal from fastBAT is orders of magnitude higher than the top associated SNP from GWAS, suggesting there are multiple independent causal variants in these regions. Shown are the results from the GCTA condition analysis using the 1KGP-imputed HRS data as the reference for LD estimation (Methods). Shown in red are the original GWAS results, in green are the results from the conditional analysis conditioning on the top SNP (labeled as ‘Cond round 1’), and in blue are the results from the conditional analysis conditioning on the top two independent signals (‘Cond round 2’). The blue horizontal line represents the threshold p-value of 5 × 10^−8^.

Of the novel genes identified by fastBAT using the latest GWAS data, 6 genes for height, 2 for BMI, and 3 for abbreviated SCZ passed the commonly used GWAS threshold p-value (i.e. *P* < 5 × 10^−8^). While a few of these results are likely due to sampling, e.g. *P*_fastBAT_ just passed the threshold whereas *P*_GWAS_ of the top associated SNP was slightly below the threshold, there were genes for which *P*_fastBAT_ was orders of magnitude smaller than *P*_GWAS_ of the top associated SNP. These include *THRB* and *FOXP1* for height, *SCAMP4* for BMI, and *FOXP1* and *ZNF365* for SCZ. We have shown by simulations above that if there is only one causal variant at a locus, *P*_fastBAT_ is expected to be larger than *P*_GWAS_ of the top associated SNP (Supplementary Fig. 1). Hence, the gain of power for fastBAT at these 5 loci is likely due to multiple signals. We therefore performed GCTA-COJO conditional analysis in these 5 gene regions (Methods). We found that there was at least a secondary (but not genome-wide significant) signal conditioning on the top associated SNP in each of these regions (Fig. 3). Interestingly, *FOXP1* was associated with both height (*P*_fastBAT_ = 1.7 × 10^−9^) and SCZ (*P*_fastBAT_ = 3.8 × 10^−12^), consistent with previous evidence that *de novo* mutations in *FOXP1* cause intellectual disability, autism, and language impairment in humans^18^, that increased gene expression level of *FOXP1* in autism patients^19^, and that *Foxp1* deletion impairs neuronal development and causes autistic-like behaviour in mice^20^.

## Discussion

We have shown above that fastBAT is equivalent to the two prevailing methods, PLINK and VEGAS, for p-values > 10^−6^, and is more accurate than both methods for very small p-value since p-values from the permutation- or simulation-based methods are bounded by the number of simulations or permutations. There is no performance penalty for fastBAT to calculate very small p-values, which allows for analyses in very large data sets (e.g. large-scale meta-analysis of GWAS) or traits for which there are genes with large effects (e.g. endophenotypes). We have implemented fastBAT with a user-friendly interface as part of the GCTA software package (http://cnsgenomics.com/software/gcta/fastBAT.html). Though the fastBAT method itself is fast, the GCTA-fastBAT implementation has been further optimized by using the efficient EIGEN library (http://eigen.tuxfamily.org) for linear algebra calculation, and by using the parallel computing technique OpenMP for multi-threaded computing. We benchmarked the computational efficiency by simulations (Supplemental Note) that the GCTA-fastBAT is orders of magnitude faster than PLINK (10^6^ permutations) and VEGAS (command-line version; 10^6^ simulations). We further demonstrated by simulations and analysis of real data that in comparison with the recently developed method Pascal (Pascal and fastBAT both belong to the same family of methods), fastBAT has greater accuracy for extremely small p-values and faster running time with lower memory requirement (Supplementary Fig. 5). GCTA-fastBAT also provides easy-to-use command that allows users to choose reference samples for LD estimation. For a single-cohort based GWAS, the GWAS cohort itself can be used as the reference sample, which further improves the accuracy as compared with using HapMap or 1KGP samples (Fig. 1d). For a meta-analysis, we can use one of the largest participating cohorts as the reference sample. GCTA-fastBAT also allows users to customize SNP sets, e.g. SNPs within genes involved in each pathway as a set. In addition, it should be noted that the sum-of-chi-squared approach implemented in fastBAT tests the average effect of all SNPs in a set, which could be less powerful than the max-of-chi-squared approach implemented in GATES and Pascal-Max, depending on the genetic architecture (the sum-of-chi-squared approach is more powerful than the max-of-chi-squared approach only if there are multiple independent signals in a set; Supplementary Fig. 2).

Using fastBAT, we analyzed data from the latest meta-analyses of GWAS for 3 complex traits and identified novel associations at 50 gene loci for height, 8 for BMI and 29 for SCZ at a genome-wide significance level (*P*_fastBAT_ < 2 × 10^−6^). These represent 1.8%, 4.8% and 3.9% of the total number of genome-wide significant genes identified for height, SCZ and BMI respectively. Of the novel associations, 6 genes for height, 2 for BMI, and 3 for abbreviated SCZ even passed the commonly used GWAS threshold p-value (i.e. *P*_fastBAT_ < 5 × 10^−8^) (Table 1). For these analyses, we used the 1KGP-imputed HRS as the reference for LD estimation (Methods). We re-ran the analyses using the 1KGP-imputed ARIC data as the reference sample (ARIC genotypes after QC were imputed to 1KGP reference panels and after imputation SNPs with HWE test p-value ≥ 1e-6, and MAF ≥ 0.01 were included in the analysis). The results were highly consistent (Supplementary Fig. 6), which again shows the robustness of the method to the sampling variation in LD estimation. We also note the results were generally robust to alternate window sizes (Supplementary Fig. 7).

In the analyses above, we investigated the properties of fastBAT and compared it with the prevailing methods using simulations based on real genotypes data from SNP-array based genotyping or whole genome sequencing (WGS). In the analyses of real data, however, we used GWAS summary data from SNP-array genotyped data being imputed to reference panels, e.g. height and BMI data from HapMap2-based imputation and SCZ data from 1KGP-based imputation. A recent study by Yang *et al*.^21^ suggests that ~97% of variation at common variants (minor allele frequency, MAF > 0.01) and ~68% variation at rare variants can be captured by imputing SNP-array genotyped data to 1KGP reference panels. These figures are interpreted as multi-variant tagging, i.e. they measure genetic variation at multiple sequence variants captured by genome-wide imputed variants. The Yang *et al*. study also reported that the single-variant tagging (squared correlation between a sequence variant and its best tagging imputed variant) is much lower, 81% for common and 25% for rare variants, for 1KGP imputation based on Illumina CoreExome arrays. We therefore performed analyses to investigate the loss of power due to imperfect imputation for fastBAT. We used the simulation strategy as described in Yang *et al*., where we extracted from the UK10K-WGS data the variants that are on Illumina CoreExome arrays, and performed 1KGP imputation based on these variants (Supplemental Note). We simulated the phenotype based on the UK10K-WGS data, and performed the fastBAT analyses using both UK10K-WGS data and the imputed data. We then calculated the correlation between −log10(*P*_fastBAT_) based on WGS data and that based on 1KGP-imputed data. On average across 100 simulation replicates, the mean *r*^2^ was 94.2% for common variants and 33.1% for rare variants (Supplementary Fig. 8). These results suggest that for set-based methods such as fastBAT, using data from 1KGP imputation is on average 94% as powerful as that using data from WGS for common variants, and 33% for rare variants, which were higher than single-variant tagging (81% for common and 25% for rare variants) but still lower than the multi-variant tagging (97% for common and 68% for rare variants) quantified by Yang *et al*. using the same data sets. This suggests that there is still a room to improve the power of the set-based test by a multivariate approach (e.g. fitting the SNP effects as random effects) using summary data (strictly speaking, fastBAT is not a multivariate method because it does not re-estimate the SNP effects in a joint model).

In summary, we propose a fast and efficient set-based association test (fastBAT) and implemented it in a user-friendly software tool (GCTA-fastBAT, http://cnsgenomics.com/software/gcta/fastBAT.html). Using this method, we identified novel associations using summary data from the latest meta-analyses of GWAS for height, BMI and abbreviated SCZ. Since the method only requires single-SNP based association p-values and a reference sample for LD estimation, it can be applied to both quantitative trait and case-control studies in humans and other species.

## Methods

### fastBAT calculation of set-based association p-value

Let **z** = {*z*_i_} be a vector of z-statistics for a set of SNPs from a GWAS or meta-analysis. Under the null hypothesis of no association between any of the SNPs and the trait, **z** follows a multivariate normal distribution, i.e. **z** ~ MVN(**0**, **R**) where **R** is the LD correlation matrix for the SNPs^7^. To test the significance of the effect sizes for a set of SNPs, PLINK set-based analysis or VEGAS uses the test-statistic *T* = Σ *z*^2^_i_, i.e. sum of chi-squared statistics of all SNPs. Since the *T* statistic does not have an explicit cumulative density function, PLINK or VEGAS calculates the gene-based p-value by contrasting the observed *T* value to an empirical distribution generated from resampling under the null hypothesis (PLINK uses permutations and VEGAS uses simulations)^7^. In fact, *T* can be expressed by a quadratic form of the vector **z**, i.e. *T* = **z**^T^**Iz** with **I** being an identity matrix. The distribution of a quadratic form of multivariate normal variables can be approximated by the Satterthwaite or Saddlepoint methods^22^,^23^ with high accuracy, as implemented in the *pchisqsum*() function in R.

### Data for method comparison

We used GWAS data on 7,663 unrelated individuals (SNP-derived genetic relatedness < 0.025) from the Atherosclerosis Risk in Communities (ARIC) study^24^ after quality controls, missingness > 0.02, MAF < 0.01, and Hardy-Weinberg Equilibrium (HWE) test p-value < 0.001. Detailed description of the cohort, SNP genotyping and QC can be found elsewhere^2^. For the ease of the permutation analysis with PLINK, we only used 7,608 SNPs on chromosome 22 for method comparison. For the analysis of real phenotype data, we used cleaned height phenotype data from a previous study (adjusting phenotype for age and sex)^2^. For the analysis using a simulated phenotype, we randomly sampled 50 SNPs from the ARIC data as causal variants, and simulated a quantitative trait using the GCTA simulation function (–simu-qt option) with each causal variant explaining 0.4% of phenotypic variance. We defined SNPs within 50 Kb from UTRs of a gene as a set. The list of genes with their start and end positions (based on hg19) is available at the PLINK website, and mirrored at the GCTA-fastBAT website. For PLINK, we used individual-level genotype and phenotype data. For VEGAS and fastBAT analyses, we used summary data from association analyses in PLINK (–assoc option) and LD between SNPs calculated from the ARIC genotypes. To investigate the robustness of VEGAS and fastBAT to the sampling variation in LD, we also performed the analyses with LD from the HapMap2 CEU samples (*n* = 90). In addition, we also simulated phenotypes without any genetic effect to investigate the inflation (or deflation) of the test-statistics for fastBAT under the null.

### Simulating unlinked SNPs with one or more causal variants

To compare the power between set-based test and single-SNP based test, we simulated genotypes of a set of *m* unlinked SNPs for 10,000 individuals, where *m* was the number of SNPs within 50 Kb from UTRs of each gene from the genotype data of the ARIC study, excluding genes with less than 10 SNPs (resulting in 450 genes). Allele frequencies of the SNPs were generated from a uniform distribution, *p* ~ U(0.01, 0.99), and the genotypes of each SNPs were generated from a binomial distribution, *x* ~ B(2, *p*). We randomly sampled *m*_c_ SNP as causal variants, and generated the effect sizes of the causal variants from a standard normal distribution, *b* ~ *N*(0, 1). The phenotypes were simulated following the method used in the GCTA simulation function, i.e. 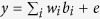 where 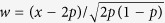 and *e* is the residual. We simulated the residual variance such that each causal variant explained 0.4% of phenotypic variance. We repeated the simulation 10 times for each of the 450 sets under two scenarios, I) one causal variant per gene, and II) three causal variants per gene.

### Simulating SNPs in perfect LD

We used the strategy as described above to simulate *m* unlinked SNPs for 10,000 individuals. For each of the 450 sets, we simulated only one causal variant per set. We then added an additional SNP to each set in two scenarios: I) adding a SNP that is in perfect LD with the causal variant, or II) adding a SNP that is in perfect LD with a non-causal SNP selected at random. We then performed fastBAT analysis using these two addition sets and compared the results with the original set.

### The LD-pruned fastBAT method

For each set of SNPs (e.g. SNPs centered around a gene), we had a correlation matrix representing LD between all SNPs. We recorded all SNP pairs with squared correlation greater than the LD *r*^2^ threshold. As one SNP might be correlated with many others, we counted the number of times each SNP appeared and used this to remove the smallest number of SNPs that still kept the pairwise correlation below the threshold. We then used the remaining SNPs for a fastBAT analysis. We call this method LD-pruned fastBAT.

We used simulations to quantify empirically the gain or loss of power for LD-pruned fastBAT as a function of the *r*^2^ threshold used for LD pruning. The simulations were based on whole genome sequencing (WGS) data from the UK10K project^25^, 1,307,127 variants (581,606 SNPs with MAF ≥ 0.01) on chromosome 1 and 3,781 individuals after QC (see Yang *et al*.^21^ for details about QC). We selected 50 common variants (MAF ≥ 0.01) as causal variants and generated quantitative phenotypes using the GCTA simulation function (–simu-qt option) with each causal variant explaining 0.2% of phenotypic variance. We simulated the causal variants using two strategies, I) causal variants were sampled completely at random, and II) causal variants were clustered in random genomic regions. To generate clusters of causal variants, we randomly selected a 200 Kb region, and then used a binomial distribution (size parameter = 50 and frequency parameter = 0.1) to determine how many of the total 50 causal variants were in this region (cluster). We repeated the procedure across the chromosome until there were multiple clusters totaling 50 causal variants.

We performed a gene-based fastBAT analysis of the simulated phenotypes with 20 different LD cutoff values, from 1 to 0.1 (at 0.1 intervals between 0.1 and 0.9, and at 0.01 intervals between 0.9 and 1) for three different SNP panels: all common sequence variants from UK10K, HapMap2 SNPs, and HapMap3 SNPs. As described above, we defined a gene-based set as the SNPs within 50 Kb from the UTRs of a gene. For the ease of calibration, we transformed *P*_fastBAT_ of each gene into a *χ*^2^ value with 1 degree of freedom. The power was measured by the mean 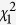 value over all the genes or the genes with a least one of the simulated causal variants. We repeated the simulation 500 times for each set of parameters, and took the average of the mean 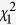 value over 500 simulations.

### fastBAT analysis using summary data from GWAS for height, BMI and SCZ

We performed a gene-based fastBAT analysis (SNPs within 50 Kb from the UTRs of a gene as a set) for height, BMI and SCZ using summary data from the latest meta-analyses of GWAS (Supplementary Table 2). We used GWAS data from the Health Retirement Study (HRS) as the reference sample for LD estimation. The HRS GWAS data were imputed to the 1KGP reference panels (Phase 3) using IMPUTE2 (ref. 26), and after imputation only the SNPs with HWE test p-value ≥ 1 × 10^−6^, and MAF ≥ 0.01 were included in the analysis.

### Conditional analysis at the novel gene loci

To detect multiple independent signals at the novel gene loci, we performed conditional analyses with GCTA-COJO^4^,^27^ using summary-data from the latest meta-analysis of GWAS for height, BMI and SCZ and LD between SNPs from a reference sample (i.e. the 1KGP-imputed HRS data). We performed an association analysis using GCTA (–cojo-cond option) conditioning on the top associated SNP (i.e. the primary signal in the region) and selected the secondary signal (i.e. the top associated SNP conditioning on the primary signal). We repeated the process to test whether there was a tertiary signal conditioning on the primary and secondary signals. Results from these conditional analyses are visualized in Fig. 3.

## Additional Information

**How to cite this article**: Bakshi, A. *et al*. Fast set-based association analysis using summary data from GWAS identifies novel gene loci for human complex traits. *Sci. Rep.* **6**, 32894; doi: 10.1038/srep32894 (2016).

## Supplementary Material

Supplementary Information

## Figures and Tables

**Table 1:** Novel gene loci identified by fastBAT at *P* < 5 × 10^−8^ for height, BMI and schizophrenia.

Trait	Chr	Gene	Top associated SNP	TOP *P*_GWAS_	*P*_fastBAT_
Height	3	THRB	rs2360960	1.20E-07	1.33E-09
Height	3	FOXP1	rs7617596	2.10E-07	1.74E-09
Height	22	UBE2L3	rs5754217	8.50E-08	6.89E-09
Height	8	RBPMS	rs2979510	5.80E-08	8.11E-09
Height	6	MIR6780B	rs2487663	1.70E-07	1.63E-08
Height	7	CALU	rs1043595	1.20E-07	2.68E-08
SCZ	3	FOXP1	rs7372960	1.25E-07	3.83E-12
SCZ	10	ZNF365	rs72829007	1.61E-07	9.32E-11
SCZ	15	AP3B2	rs113272695	1.70E-07	2.51E-08
BMI	19	SCAMP4	rs11672550	1.33E-07	9.77E-10
BMI	7	DTX2P1-UPK3BP1-PMS2P11	rs6955651	6.19E-08	2.43E-08

Top *P*_GWAS_: p-value of the top associated SNP in GWAS.
