# Supplementary material for: Fast set-based association analysis using summary data from GWAS identifies novel gene loci for human complex traits
Source: Sci Rep. 2016 Sep 8;6:32894. doi: 10.1038/srep32894 (PMC5015118; doi:10.1038/srep32894)
Supplement: Supplementary Information [file srep32894-s1.pdf]

**Supplementary Online Material**

# Fast set-based association analysis using summary data from GWAS identifies novel gene loci for human complex traits

Andrew Bakshi[1], Zhihong Zhu[1], Anna A.E. Vinkhuyzen[1], W. David. Hill[2,3], Allan F. McRae[1], Peter M. Visscher[1,4] and Jian Yang[1,4]

1. Queensland Brain Institute, The University of Queensland, Brisbane, Queensland 4072, Australia
2. Centre for Cognitive Ageing and Cognitive Epidemiology, University of Edinburgh, 7 George Square, Edinburgh, UK
3. Department of Psychology, University of Edinburgh, Edinburgh, UK
4. The University of Queensland Diamantina Institute, The Translation Research Institute, Brisbane, Queensland, Australia

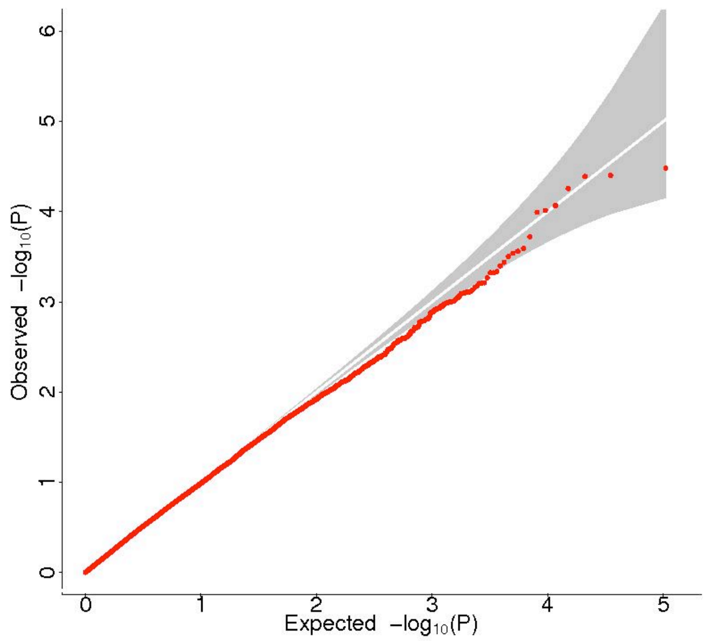


**Supplementary Figure 1** QQ plot of $P_{\text{fastBAT}}$ from simulations under the null. The simulation is based observed SNP genotypes from the ARIC cohort (Online Methods). Shown is the QQ plot of p-values from 100 simulations (526 gene sets × 100 simulation replicates) using fastBAT. Mean $\chi^2{}_1$ value across 100 simulations = 0.9993 (standard error of the mean, s.e.m = 0.015), where $\chi^2{}_1$ is calculated from $P_{\text{fastBAT}}$.

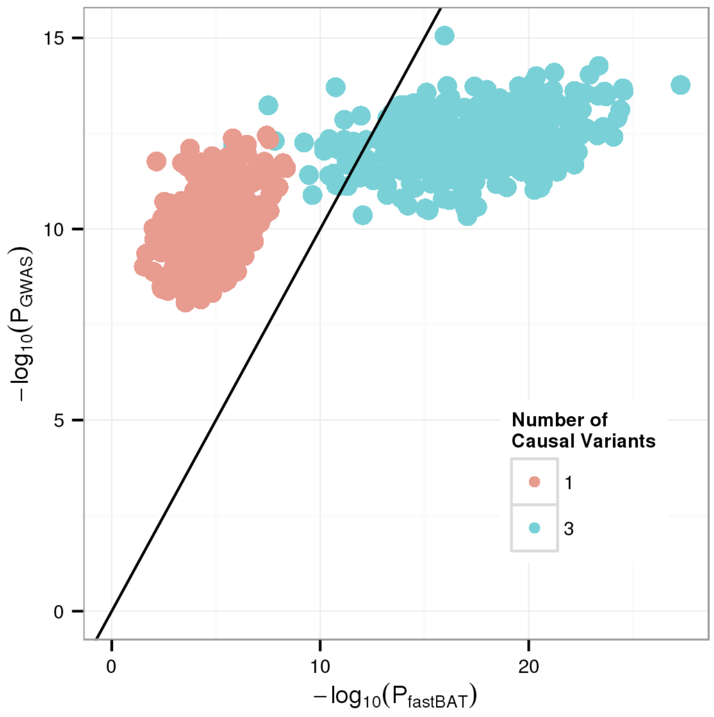


**Supplementary Figure 2** Comparison between single-SNP and set-based tests**.** Results are from simulations of unlinked SNPs in 450 sets (Online Methods), each causal variant explaining 0.4% of phenotypic variance. Shown on the y-axis is the –log10(p-value) of the top associated SNP in a set and shown on the x-axis is the –log10(p-value) from the fastBAT test using all SNPs of the set. Each dot represents the average from 10 simulation replicates.

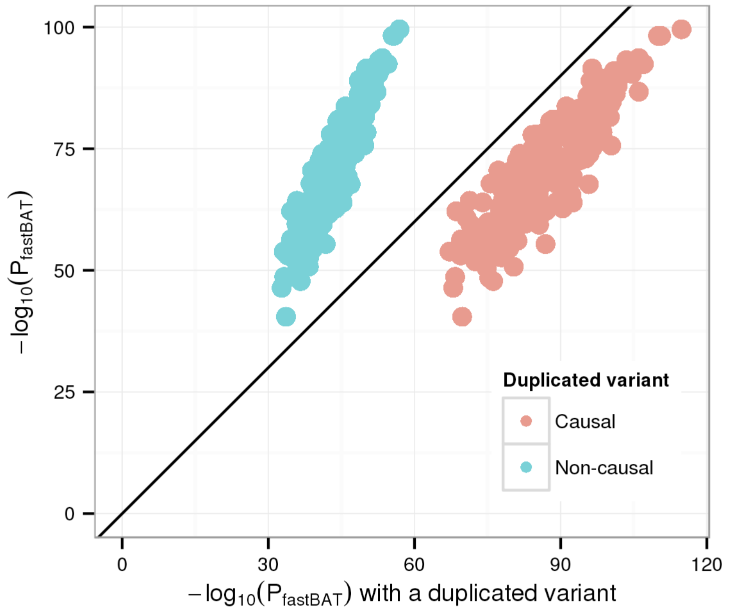


**Supplementary Figure 3** The change of power by duplicating a variant in a set. Results are from simulations of unlinked SNPs in 450 sets (Online Methods). There is only one causal variant (explaining 0.4% of phenotypic variant) in each set. Shown is the –log10(p-value) from the fastBAT analysis with the original set plotted against that with the an additional variant in perfect LD with the causal (in red) or non-causal (in blue) in the original set. Each dot represents the average from 10 simulation replicates.

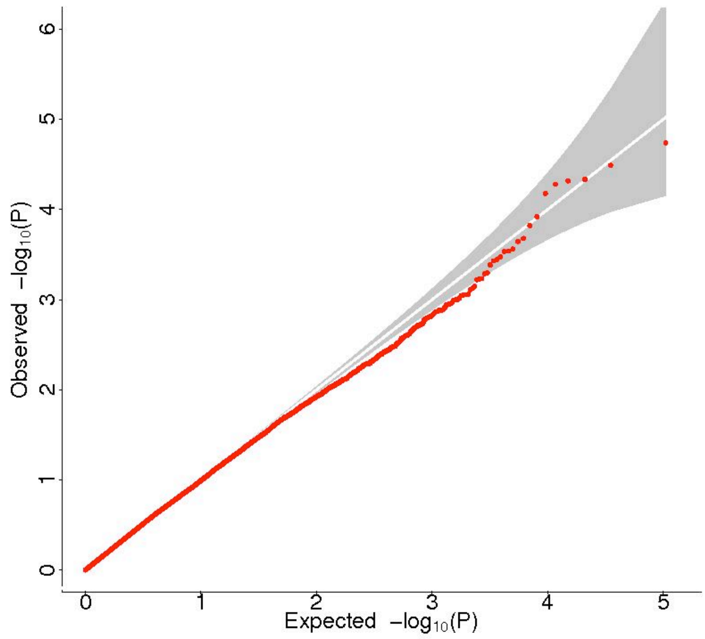


**Supplementary Figure 4** QQ plot of $P_{\text{fastBAT}}$ from simulations under the null. Shown is the QQ plot of p-values from LD-pruned fastBAT ($r^2$ threshold of 0.9) analyses of data simulated in **Supplementary Figure 1**. Mean $\chi^2_1$ value across 100 simulations = 0.9996 (s.e.m. = 0.015), where $\chi^2_1$ is calculated from $P_{\text{fastBAT}}$.

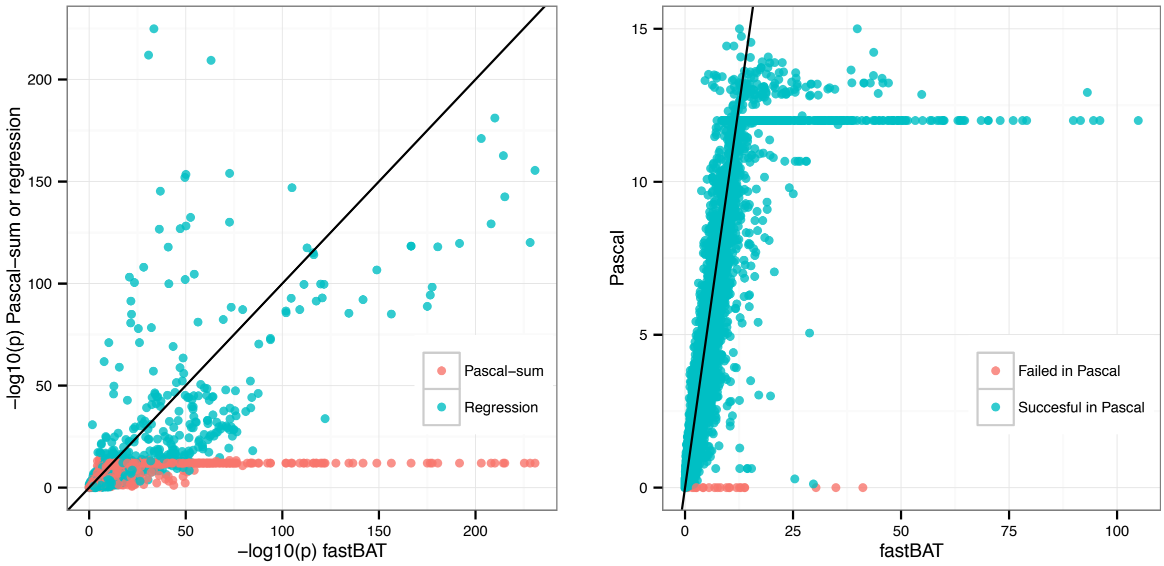


**Supplementary Figure 5** Comparison between fastBAT and Pascal using simulations and analysis of real data. Shown are the results from gene-based analyses using the latest versions of fastBAT (GCTA version 1.25.2) and Pascal (downloaded on 27 Jan 2016) with default options. A gene region is defined as ±50Kb of UTRs. We used the 1KGP-EUR data as the reference set for LD estimation. For Pascal analysis, we used the 1KGP-EUR data in Pascal format, which were downloaded as part of the Pascal package. For fastBAT analysis, we used the 1KGP-EUR data (Phase 3; SNP inclusion criteria: missingness rate < 0.05, HWE p > $1 \times 10^{-6}$ and MAF > 0.0025) downloaded from 1KGP website (**URLs**). In panel (a), the GWAS data were generated from 50 simulations based on real genotypes. In each simulation replicate, we randomly sampled 10 common SNPs (MAF ≥ 0.01) on chromosome 22 as causal variants from the 1KGP-imputed GERA study[1] ($n$ = 53,991 unrelated individuals of European ancestry), and generated a phenotype based on the simulated causal variants, where each causal variant explains 0.4% of phenotypic variation. Regression: linear regression analysis of phenotype on causal variant(s) in each gene region, which is is used as the gold standard for the comparison between fastBAT and Pascal. In panel (b), we used the GWAS summary data from the GIANT meta-analysis for height[2]. There were 30 genes (highlighted in red) for which Pascal could not determine a p-value. It shows in both panels (a) and (b) that p-values from Pascal are bounded at about $1 \times 10^{-15}$. This might not be an issue for gene discovery but could potentially be a problem to prioritise the top associated genes for follow-up functional studies. fastBAT also outperforms Pascal in computational efficiency, e.g. for the analysis of height data, fastBAT took 40 sec using 302MB memory, and Pascal took 502 sec using 5,152MB memory (mean values quantified from 50 repeats on identical hardware).

a) Height

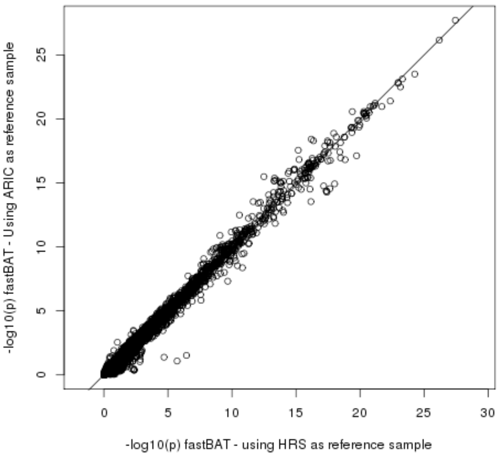


b) BMI

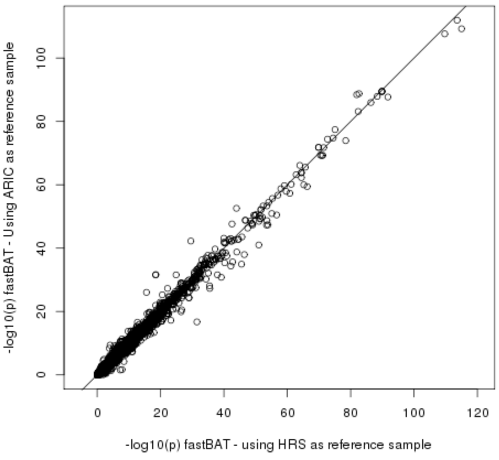


c) Schizophreina

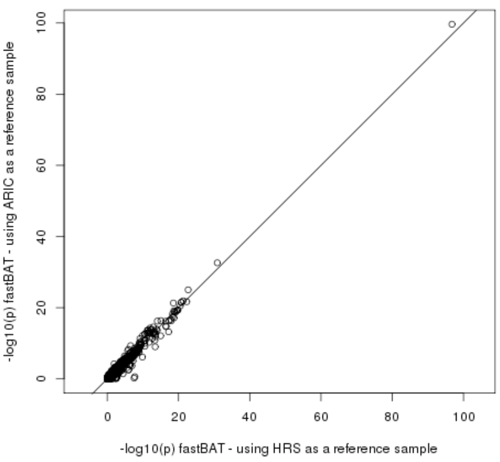


**Supplementary Figure 6** Robustness of fastBAT to variation in LD. Shown are the results from the fastBAT analysis of the latest GWAS data using the 1KGP-imputed ARIC data as a reference for LD estimation vs. that using the 1KGP-imputed HRS data for (a) height, (b) BMI, and (c) schizophrenia.

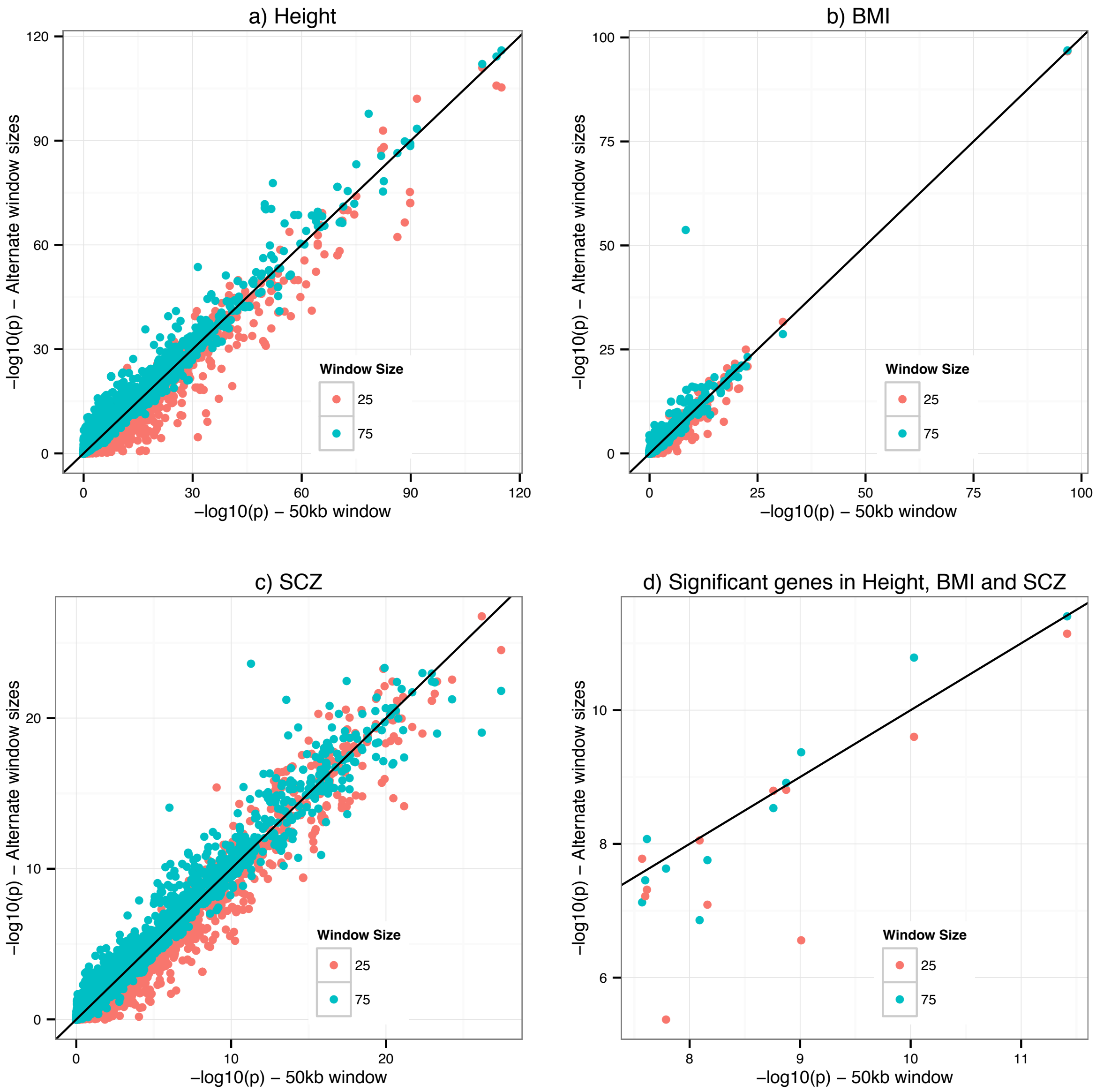


**Supplementary Figure 7** Analyses of height, BMI and schizophrenia data with different gene window sizes. In all panels the x-axis shows the -log10(p-value) calculated by fastBAT for a gene using the default window size, i.e. ±50kb of the UTRs. On the y-axis, shown are the –log10(p-value) calculated by fastBAT for the alternate window sizes of 25kb and 75kb. The first three panels show the results of all genes for (a) height, (b) schizophrenia and (c) BMI, respectively. Panel (d) is a combined set of the novel genes for three traits that passed the commonly used GWAS threshold p-value ($P_{\text{fastBAT}} < 5\times10^{-8}$) with a 50kb window (**Table 1**), and their respective p-values with alternate windows. All but one of the novel genes were still significant to a genome-wide significance level ($P_{\text{fastBAT}} < 2\times10^{-6}$) with the alternate window sizes.

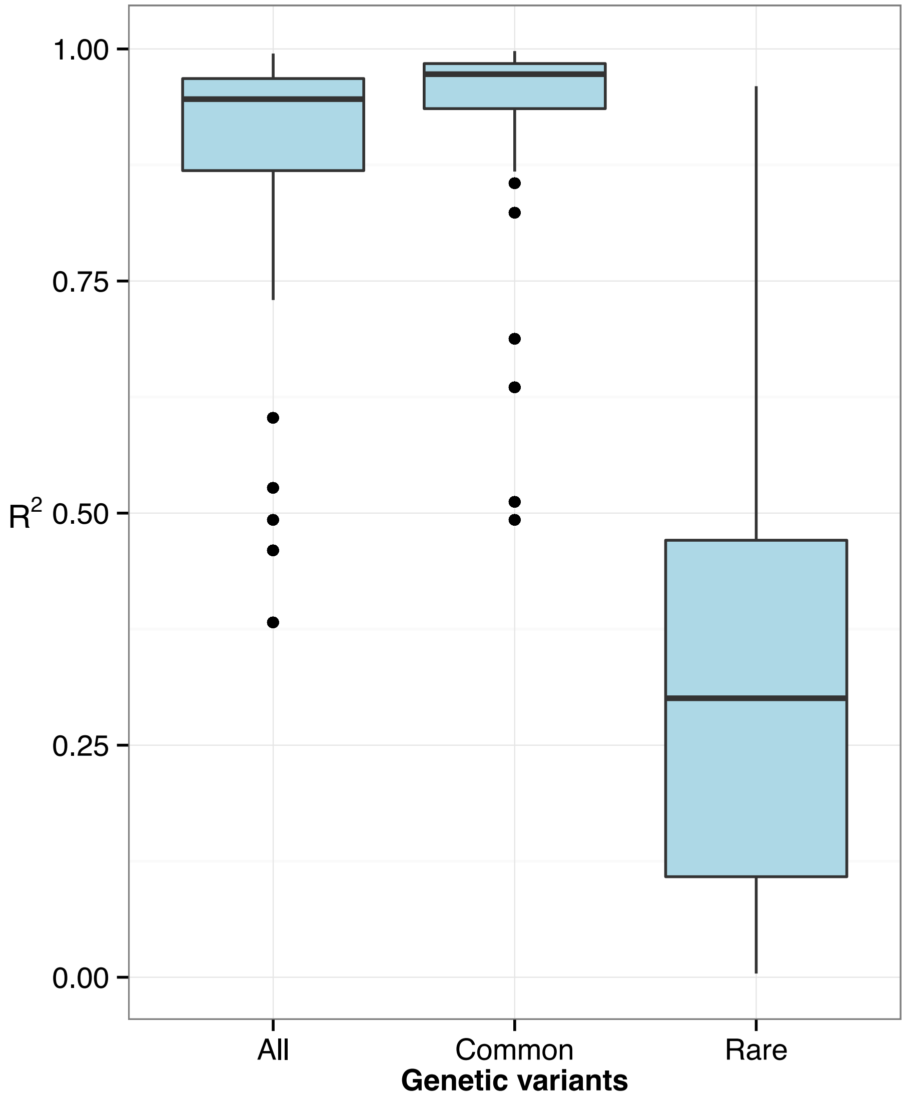


**Supplementary Figure 8** Correlation between –log10($P_{\text{fastBAT}}$) using WGS data and that using 1KGP-imputed data. Shown are the results from simulations based on UK10K-WGS data (**Supplemental Note**). In each simulation replicate, a quantitative trait is simulated using WGS data, and the analysis was performed using both WGS and 1KGP-imputed data (see **Supplemental Note** for details). Common: variants with MAF ≥ 0.01. Rare: variants with MAF < 0.01.

**Supplementary Table 1** Mean $\chi^2_1$ values calculated from fastBAT analysis with and without LD pruning using simulations. The simulations are based on UK10K-WGS data (Online Methods). The $\chi^2_1$ value is calculated from $P_{\text{fastBAT}}$. The mean $\chi^2_1$ value is calculated from 500 simulation replicates.

| Distribution of causal variants | No LD pruning | | LD pruning with $r^2$ threshold = 0.99 | | LD pruning with $r^2$ threshold = 0.9 | |
|---|---|---|---|---|---|---|
| | **All** | **Causal** | **All** | **Causal** | **All** | **Causal** |
| Random | 1.216 | 4.27 | 1.229 | 4.34 | 1.235 | 4.13 |
| Clustered | 1.229 | 12.72 | 1.247 | 13.34 | 1.261 | 13.41 |

**Supplementary Table 2** Descriptive summary of the GWAS summary data

| Version | Trait | Sample size | Number of SNPs | Reference |
|---|---|---|---|---|
| **Latest** | Height | 253,288 | 2.6M | Wood et al. 2014 Nature Genetics[2] |
| | BMI | 339,224 | 2.6M | Locke et al. 2015 Nature[3] |
| | SCZ | 36,989 vs. 113,075 | 9.4M | PGC 2014 Nature[4] |
| **Earlier** | Height | 133,653 | 2.7M | Lango Allen et al. 2010 Nature[5] |
| | SCZ | 9,394 vs. 12,462 | 1.3M | PGC 2011 Nature Genetics [6] |

**Supplementary Table 3** "Novel" loci identified by fastBAT analyses using the earlier version of GWAS data for height and schizophrenia. "earlier": the earlier version of GWAS data (**Supplementary Table 2**). "latest": the latest version of GWAS data (**Supplementary Table 2**). "Top $P_{GWAS}$": p-value of the top associated SNP in GWAS.

| Trait | Chr | Gene | Top $P_{GWAS}$ (earlier) | $P_{fastBAT}$ (earlier) | Top $P_{GWAS}$ (latest) | $P_{fastBAT}$ (latest) |
|---|---|---|---|---|---|---|
| SCZ | 1 | SDCCAG8 | 3.26E-06 | 1.45E-06 | 3.73E-09 | 1.46E-10 |
| SCZ | 3 | MUSTN1 | 2.25E-06 | 1.10E-06 | 4.26E-11 | 2.71E-11 |
| SCZ | 7 | MAD1L1 | 5.06E-08 | 5.41E-08 | 6.43E-14 | 5.27E-11 |
| SCZ | 12 | CACNA1C | 5.06E-08 | 1.88E-07 | 3.217E-18 | 2.06E-18 |
| Height | 1 | SKI | 3.07E-06 | 1.51E-06 | 3.30E-17 | 2.16E-19 |
| Height | 2 | BOK-AS1 | 7.05E-06 | 2.49E-07 | 1.40E-12 | 1.02E-16 |
| Height | 3 | HDAC11 | 1.15E-07 | 1.04E-06 | 3.10E-16 | 5.41E-15 |
| Height | 3 | SHOX2 | 4.87E-07 | 4.72E-07 | 9.70E-13 | 3.03E-11 |
| Height | 3 | ZBTB20 | 4.49E-06 | 4.19E-07 | 6.60E-11 | 2.20E-16 |
| Height | 4 | TACC3 | 6.02E-08 | 1.91E-07 | 1.80E-18 | 6.06E-20 |
| Height | 7 | RPS2P32 | 5.56E-08 | 4.90E-08 | 3.50E-26 | 1.96E-28 |
| Height | 8 | ENPP2 | 1.29E-06 | 8.33E-07 | 3.50E-13 | 1.05E-16 |
| Height | 11 | SENCR | 6.75E-07 | 4.89E-07 | 2.70E-14 | 1.35E-14 |
| Height | 11 | TEAD1 | 6.07E-08 | 1.46E-07 | 1.10E-15 | 1.28E-16 |
| Height | 12 | CCDC53 | 1.09E-07 | 4.18E-08 | 1.50E-19 | 1.42E-20 |
| Height | 14 | RAD51B | 3.99E-06 | 2.20E-07 | 3.80E-14 | 2.81E-17 |
| Height | 16 | HAGHL | 2.40E-07 | 5.87E-08 | 1.40E-18 | 4.56E-20 |
| Height | 17 | GIT1 | 1.58E-06 | 3.97E-07 | 1.10E-12 | 3.50E-13 |
| Height | 17 | KCNJ12 | 2.00E-07 | 3.20E-08 | 8.40E-14 | 9.43E-14 |
| Height | 17 | UBE2Z | 1.86E-07 | 1.93E-06 | 1.50E-16 | 7.10E-15 |
| Height | 19 | ADAMTS10 | 2.52E-07 | 6.80E-07 | 1.40E-18 | 1.17E-18 |
| Height | 19 | INSR | 1.72E-06 | 7.76E-08 | 7.20E-18 | 7.65E-22 |
| Height | 19 | NFIC | 2.25E-07 | 6.53E-08 | 1.30E-20 | 1.83E-25 |

**Supplementary Table 4** Novel gene loci identified by fastBAT for height, BMI and schizophrenia at a genome-wide significance level ($P$ < 2e-6). "Top $P_{GWAS}$": p-value of the top associated SNP in GWAS.

| Trait | Chr | Gene | Top associated SNP | Top $P_{GWAS}$ | $P_{fastBAT}$ |
|---|---|---|---|---|---|
| Height | 3 | THRB | rs2360960 | 1.20E-07 | 1.33E-09 |
| Height | 3 | FOXP1 | rs7617596 | 2.10E-07 | 1.74E-09 |
| Height | 22 | UBE2L3 | rs5754217 | 8.50E-08 | 6.89E-09 |
| Height | 8 | RBPMS | rs2979510 | 5.80E-08 | 8.11E-09 |
| Height | 6 | MIR6780B | rs2487663 | 1.70E-07 | 1.63E-08 |
| Height | 7 | CALU | rs1043595 | 1.20E-07 | 2.68E-08 |
| Height | 2 | PCBP1 | rs6546568 | 1.20E-07 | 6.23E-08 |
| Height | 2 | COL6A3 | rs6719451 | 3.30E-07 | 6.36E-08 |
| Height | 19 | APOC4 | rs2288911 | 2.80E-07 | 7.40E-08 |
| Height | 7 | AUTS2 | rs10262697 | 2.70E-07 | 7.93E-08 |
| Height | 1 | TIPRL | rs10737541 | 5.50E-07 | 9.86E-08 |
| Height | 20 | TOMM34 | rs2180292 | 9.30E-07 | 1.67E-07 |
| Height | 1 | CNIH4 | rs12754832 | 5.50E-08 | 1.79E-07 |
| Height | 8 | TOX | rs3780001 | 1.50E-06 | 1.95E-07 |
| Height | 16 | WWOX | rs4444350 | 1.40E-07 | 2.04E-07 |
| Height | 11 | SYTL2 | rs290195 | 8.30E-08 | 2.46E-07 |
| Height | 3 | MECOM | rs2014590 | 6.40E-07 | 3.02E-07 |
| Height | 1 | RBM34 | rs4551650 | 3.90E-07 | 3.02E-07 |
| Height | 7 | KDM7A | rs7797205 | 8.00E-08 | 3.13E-07 |
| Height | 12 | C12orf10 | rs7398691 | 1.30E-07 | 3.31E-07 |
| Height | 6 | KIAA1586 | rs720884 | 6.00E-07 | 3.59E-07 |
| Height | 17 | ZNF18 | rs7216812 | 1.40E-06 | 3.80E-07 |
| Height | 18 | PSMA8 | rs4800724 | 7.60E-07 | 3.83E-07 |
| Height | 12 | E2F7 | rs310796 | 2.80E-07 | 4.13E-07 |
| Height | 22 | SCUBE1 | rs998409 | 1.50E-06 | 5.16E-07 |
| Height | 6 | STX7 | rs7743622 | 7.10E-08 | 5.48E-07 |
| Height | 2 | ATOH8 | rs1465821 | 7.80E-07 | 5.82E-07 |
| Height | 5 | SRFBP1 | rs12153375 | 2.20E-07 | 6.02E-07 |
| Height | 16 | TEKT5 | rs8057807 | 8.50E-08 | 6.03E-07 |
| Height | 20 | MYBL2 | rs387769 | 8.00E-06 | 6.18E-07 |
| Height | 19 | ARHGAP33 | rs2280743 | 1.50E-06 | 6.23E-07 |
| Height | 4 | SLC7A11 | rs4863767 | 1.30E-06 | 6.86E-07 |
| Height | 22 | TUG1 | rs5749202 | 5.20E-08 | 7.31E-07 |
| Height | 13 | LINC00462 | rs12871822 | 5.70E-08 | 7.43E-07 |
| Height | 4 | LIMCH1 | rs11726922 | 1.20E-07 | 7.71E-07 |
| Height | 8 | ZHX2 | rs4128589 | 6.30E-07 | 7.80E-07 |
| Height | 10 | DLG5 | rs1248690 | 2.20E-07 | 8.65E-07 |
| Height | 8 | TMEM74 | rs7007200 | 8.80E-08 | 9.12E-07 |
| Height | 6 | TRAM2 | rs614570 | 1.90E-06 | 9.19E-07 |
| Height | 17 | MED9 | rs7946 | 8.90E-07 | 9.73E-07 |
| Height | 2 | LOC101060091 | rs6542180 | 1.20E-07 | 1.16E-06 |

| | | | | | |
|---|---|---|---|---|---|
| Height | 2 | LOC101927619 | rs2058051 | 1.30E-07 | 1.21E-06 |
| Height | 12 | WBP11 | rs16910259 | 1.10E-06 | 1.21E-06 |
| Height | 9 | FBXW2 | rs4836831 | 3.20E-07 | 1.36E-06 |
| Height | 3 | ALCAM | rs9288807 | 1.10E-07 | 1.47E-06 |
| Height | 8 | SLC45A4 | rs11167042 | 1.10E-07 | 1.51E-06 |
| Height | 4 | LOC101927007 | rs4447843 | 9.90E-07 | 1.55E-06 |
| Height | 3 | FAM198A | rs865842 | 1.90E-07 | 1.55E-06 |
| Height | 7 | CHCHD3 | rs12537090 | 1.10E-06 | 1.65E-06 |
| Height | 2 | LY75 | rs1344632 | 2.30E-07 | 1.75E-06 |
| SCZ | 3 | FOXP1 | rs7372960 | 1.25E-07 | 3.83E-12 |
| SCZ | 10 | ZNF365 | rs72829007 | 1.61E-07 | 9.32E-11 |
| SCZ | 15 | AP3B2 | rs113272695 | 1.70E-07 | 2.51E-08 |
| SCZ | 3 | RBMS3 | rs1506297 | 2.66E-07 | 5.86E-08 |
| SCZ | 18 | KCNG2 | rs56775891 | 2.17E-07 | 9.66E-08 |
| SCZ | 16 | CPNE7 | rs34753377 | 1.06E-07 | 9.75E-08 |
| SCZ | 17 | RPTOR | rs8066384 | 1.12E-06 | 1.13E-07 |
| SCZ | 8 | WHSC1L1 | rs112537273 | 3.70E-07 | 1.13E-07 |
| SCZ | 21 | DOPEY2 | rs2284641 | 1.13E-05 | 1.73E-07 |
| SCZ | 8 | ZDHHC2 | rs17687067 | 1.14E-07 | 2.36E-07 |
| SCZ | 2 | DLX1 | rs1001780 | 8.07E-06 | 3.40E-07 |
| SCZ | 17 | LOC388436 | rs4273100 | 7.77e-07 | 3.53e-07 |
| SCZ | 2 | MRPL33 | rs12474906 | 1.01E-07 | 3.56E-07 |
| SCZ | 5 | ADAMTS2 | rs1826864 | 8.44E-05 | 3.76E-07 |
| SCZ | 8 | PSD3 | rs6984438 | 2.66E-07 | 4.81E-07 |
| SCZ | 1 | CNTN2 | rs16937 | 8.69E-07 | 6.42E-07 |
| SCZ | 19 | SLC39A3 | rs57549656 | 1.22E-06 | 6.75E-07 |
| SCZ | 10 | SORCS3 | rs11192193 | 2.54E-06 | 1.03E-06 |
| SCZ | 9 | FAM120A | rs12554020 | 3.96E-06 | 1.04E-06 |
| SCZ | 11 | OTUB1 | rs571171 | 1.93E-06 | 1.18E-06 |
| SCZ | 9 | KDM4C | rs2026714 | 1.88E-07 | 1.37E-06 |
| SCZ | 8 | MSRA | rs73191547 | 9.05E-08 | 1.38E-06 |
| SCZ | 4 | CXXC4 | rs2905627 | 5.17E-07 | 1.42E-06 |
| SCZ | 2 | BCL11A | rs7599488 | 3.11E-07 | 1.44E-06 |
| SCZ | 16 | NMRAL1 | rs6500602 | 2.79E-07 | 1.57E-06 |
| SCZ | 7 | CALN1 | rs1914395 | 5.20E-07 | 1.60E-06 |
| SCZ | 15 | FAM214A | rs4776059 | 1.06E-05 | 1.89E-06 |
| SCZ | 14 | RTN1 | rs12431410 | 4.22E-07 | 1.91E-06 |
| SCZ | 2 | ERBB4 | rs16846200 | 1.62E-05 | 1.92E-06 |
| BMI | 19 | SCAMP4 | rs11672550 | 1.33E-07 | 9.77E-10 |
| BMI | 7 | DTX2P1-UPK3BP1-PMS2P11 | rs6955651 | 6.19E-08 | 2.43E-08 |
| BMI | 17 | TACO1 | rs8075273 | 1.55E-07 | 2.04E-07 |
| BMI | 9 | TRUB2 | rs2270204 | 3.22E-07 | 5.30E-07 |
| BMI | 7 | CALCR | rs9641123 | 1.83E-07 | 7.57E-07 |
| BMI | 17 | CBX1 | rs3764400 | 5.54E-07 | 8.86E-07 |
| BMI | 2 | USP37 | rs7607369 | 1.10E-07 | 1.08E-06 |
| BMI | 19 | ZNF536 | rs33439 | 7.58E-07 | 1.27E-06 |

**Supplementary Table 5** Numbers of "novel" gene loci discovered by fastBAT – with and without LD-pruning – as well as Pascal-Sum and Pascal-Max using the earlier version of GWAS data for height and schizophrenia. A "novel" gene is "replicated" if p-value of the top associated SNP in the gene region is < 5e-8 in the latest GWAS data. fastBAT analyses were performed using the 1KGP-imputed HRS as the reference sample for LD, and Pascal analyses were performed with 1KG-EUR data set provided in the Pascal software package.

| | **Height** | | **SCZ** | |
|---|---|---|---|---|
| | Discovered | Replicated | Discovered | Replicated |
| **fastBAT with LD pruning (default)** | 19 | 19 | 4 | 4 |
| **fastBAT without LD pruning** | 16 | 16 | 4 | 4 |
| **Pascal-Sum (default)** | 15 | 15 | 4 | 3 |
| **Pascal-Max** | 10 | 9 | 3 | 1 |

## Supplemental Note

### 1. Comparison between fastBAT with sequence and imputed variants

We performed simulations based on WGS data from the UK10K project[7], of which there were 17.6M genetic variants across 3,781 unrelated individuals after quality control[8]. Following the strategy proposed in Yang et al.[8], we extracted the set of SNPs that can be found on an Illumina CoreExome array from the UK10K data, and used IMPUTE2 (ref[9]) to impute the subset of SNPs to 1000 Genome Project (1KGP) reference panels[10]. The imputed SNPs with Hardy-Weinberg Equilibrium (HWE) test p-value < 1e-6 or minor allele counts < 3 were removed from the analysis.

Following Yang et al.[8], to quantify the variation at sequence variants that can be captured by 1KGP-imputed variants, we simulated traits using WGS data and performed the analysis using 1KGP-imputed data. For the ease of computation, we only used data on chromosome 1. We simulated a quantitative trait using the GCTA simulation function (50 causal variants with a total heritability of 10%) under two scenarios, I) causal variants were sampled at random from all the sequence variants; II) causal variants are clustered in a few randomly sampled genomic regions (see Online Method for the method to simulated clustered causal variants). We then performed the fastBAT analysis for the simulated trait using the 1KGP-imputed genotypes and compared the result with that using WGS data.

### 2. Benchmarking Performance

We compared the computational performance of the three implementations, PLINK-set, VEGAS (offline version) and GCTA-fastBAT by re-running the analysis presented in **Fig. 1a** on identical hardware, recording the execution time and maximum memory usage, and reported the mean results of 10 executions. We ran a gene-based PLINK-set test ($10^6$ permutations) with the individual-level genotype and phenotype data in the ARIC cohort (chromosome 22). The GCTA-fastBAT and VEGAS (command-line version) analyses were

performed using the summary statistics. On average, PLINK-set used ~38 hours to complete the analysis (note that the set-based test implemented in PLINK2 is much faster than PLINK-set but still much slower than fastBAT), VEGAS (default parameters) took 36 minutes, and GCTA-fastBAT (using only a single thread) completed in 8 seconds (see the table below). The LD-pruned fastBAT analysis has slightly higher memory requirements than that without LD-pruning but it is still orders magnitude faster than PLINK-set and VEGAS (see the table below).

| | **Time** | **RAM** |
|---|---|---|
| PLINK-set | 38 hours | 10GB |
| VEGAS | 36 min | 1.2GB |
| fastBAT | 8.6 sec | 48MB |
| fastBAT with LD pruning | 7.9 sec | 424MB |

**3. Running fastBAT**

A complete manual is available at the GCTA website (**URLs**). The implementation of fastBAT in GCTA uses a PLINK binary file as the reference set for LD estimation. If no reference for LD is available it is possible to use the HapMap3 or 1KGP data (**URLs**). A list of gene coordinates is available from the PLINK website (**URLs**) and mirrored on the GCTA website (**URLs**).

**Acknowledgements**

**ARIC:** The Atherosclerosis Risk in Communities Study is carried out as a collaborative study supported by National Heart, Lung, and Blood Institute contracts (HHSN268201100005C, HHSN268201100006C, HHSN268201100007C, HHSN268201100008C, HHSN268201100009C, HHSN268201100010C, HHSN268201100011C, and HHSN268201100012C), R01HL087641, R01HL59367 and R01HL086694; National HumanGenome Research Institute contract U01HG004402; and National Institutes of Health contract HHSN268200625226C. The authors thank the staff and participants of the ARIC study for their important contributions. Infrastructure was partly supported by Grant Number UL1RR025005, a component of the National Institutes of Health and NIH Roadmap for Medical Research.

**HRS:** HRS is supported by the National Institute on Aging (NIA U01AG009740). The genotyping was funded separately by the National Institute on Aging (RC2 AG036495, RC4 AG039029). Genotyping was conducted by the NIH Center for Inherited Disease Research (CIDR) at Johns Hopkins University. Genotyping quality control and final preparation of the data were performed by the Genetics Coordinating Center at the University of Washington.

**GERA data:** Data came from a grant, the Resource for Genetic Epidemiology Research in Adult Health and Aging (RC2 AG033067; Schaefer and Risch, PIs) awarded to the Kaiser Permanente Research Program on Genes, Environment, and Health (RPGEH) and the UCSF Institute for Human Genetics. The RPGEH was supported by grants from the Robert Wood Johnson Foundation, the Wayne and Gladys Valley Foundation, the Ellison Medical Foundation, Kaiser Permanente Northern California, and the Kaiser Permanente National and Northern California Community Benefit Programs.

**UK10K:** The UK10K project was funded by the Wellcome Trust award WT091310. Twins UK (TUK): TUK was funded by the Wellcome Trust and ENGAGE project grant agreement HEALTH-F4-2007–201413. The study also receives support from the Department of Health via the National Institute for Health Research (NIHR)-funded BioResource, Clinical Research Facility and Biomedical Research Centre based at Guy's and St. Thomas' NHS Foundation Trust in partnership with King's College London. Dr Spector is an NIHR senior Investigator and ERC Senior Researcher. Funding for the project was also provided by the British Heart Foundation grant PG/12/38/29615 (Dr Jamshidi). A full list of the investigators who contributed to the UK10K sequencing is available from www.UK10K.org.

## References


1. Banda, Y. *et al.* Characterizing Race/Ethnicity and Genetic Ancestry for 100,000 Subjects in the Genetic Epidemiology Research on Adult Health and Aging (GERA) Cohort. *Genetics* **200**, 1285-95 (2015).
2. Wood, A.R. *et al.* Defining the role of common variation in the genomic and biological architecture of adult human height. *Nat. Genet.* **46**, 1173-1186 (2014).
3. Locke, A.E. *et al.* Genetic studies of body mass index yield new insights for obesity biology. *Nature* **518**, 197-206 (2015).
4. Schizophrenia Working Group of the Psychiatric Genomics Consortium. Biological insights from 108 schizophrenia-associated genetic loci. *Nature* **511**, 421-7 (2014).
5. Lango Allen, H. *et al.* Hundreds of variants clustered in genomic loci and biological pathways affect human height. *Nature* **467**, 832-838 (2010).
6. Schizophrenia Psychiatric Genome-Wide Association Study, C. Genome-wide association study identifies five new schizophrenia loci. *Nat. Genet.* **43**, 969-976 (2011).
7. The UK10K Consortium. The UK10K project identifies rare variants in health and disease. *Nature* **526**, 82-90 (2015).
8. Yang, J. *et al.* Genetic variance estimation with imputed variants finds negligible missing heritability for human height and body mass index. *Nat. Genet.* **47**, 1114-1120 (2015).
9. Howie, B., Fuchsberger, C., Stephens, M., Marchini, J. & Abecasis, G.R. Fast and accurate genotype imputation in genome-wide association studies through pre-phasing. *Nat. Genet.* **44**, 955-959 (2012).
10. Delaneau, O., Marchini, J., Genomes Project, C. & Genomes Project, C. Integrating sequence and array data to create an improved 1000 Genomes Project haplotype reference panel. *Nat Commun* **5**, 3934 (2014).
